# High variation in the gluten composition and grain protein content among synthetic wheat lines

**DOI:** 10.1371/journal.pone.0331619

**Published:** 2025-10-10

**Authors:** Rahman Ebrahimzadegan, Ali Sanati, Ghader Mirzaghaderi

**Affiliations:** Department of Plant Production and Genetics, Faculty of Agriculture, University of Kurdistan, Sanandaj, Iran; Institute of Genetics and Developmental Biology Chinese Academy of Sciences, CHINA

## Abstract

Wheat wild relatives are important sources for the genetic enhancement of cultivated wheat. Here, we evaluated the gluten composition, grain protein content, and several quality-related gluten indices across 47 synthetic wheat lines or amphiploids resulted from the crosses between emmer wheat, durum wheat, *T. timopheevii*, *Ae. crassa*, *Ae. ventricosa* and *Ae. tauschii*. The grain protein content ranged from 15% to 23.5%, in 79% of the studied lines. Lines exhibiting high protein contents generally demonstrated normal gluten strength. This characteristic primarily resulted from the inclusion of emmer wheat, durum wheat, or *T. timopheevii* as one of the parental lines in their pedigree. About 18% of the lines, which mainly resulted from (*T. durum* × *Ae. tauschii*) × common wheat crosses demonstrated strong gluten properties. The analysis of high molecular weight glutenin subunits (HMW-GSs) revealed a greater diversity for the *Glu-B1* locus than those from *Glu-A1* and *Glu-D1*. The most frequently identified HMW-GSs included Null, 1, and 2* at the *Glu-A1* locus; 21 + 19, 7 + 8, 14 + 15, 6 + 8, 14 + 18, 21 + 15, 13 + 16 + 9, and 6 + 22 at the *Glu-B1*; and 3 + 10 or 3 + 10.5, 2 + 12 or 2 + 12.5, and 5 + 10 or 5 + 10.5 at the *Glu-D1*. Subunits associated with the bread-making quality of wheat, particularly observed in durum wheat × *Ae. tauschii* cross combinations. Cluster analysis based on gliadin and glutenin subunits did not accurately reflect the genomic composition of the lines, though some lines with similar genomic backgrounds were clustered together. These results suggest the potential of our synthetic wheat lines to enhance the nutritional and baking quality of wheat flour.

## Introduction

Bread wheat (*Triticum aestivum* L.) is a hexaploid species (2*n* = 6*x* = 42; AABBDD genomes) and represents the most widely cultivated staple crop in the world. It serves as a major source of calories and proteins in the human diet, despite its relatively low grain protein content (GPC), which typically ranges from 8% to 15% depending on the cultivar [1]. Wheat proteins include seed storage proteins (gluten proteins) and structural proteins (non-gluten proteins). Gluten characteristics allow for the assessment of baking quality even among wheat genotypes with similar protein contents, thereby revealing differences in protein strength among different lines [2–4]. Gluten is predominantly localized in the seed endosperm. In contrast, non-gluten fractions are specifically localized in the embryo and aleurone layers. Gluten is further classified into polymeric glutenins and monomeric gliadins, based on their solubility in alcohol-water and acid solutions, respectively. Glutenins which contribute to the dough elasticity and strength [5], are classified into high molecular weight (70–90 kDa) and low molecular weight (20–40 kDa) glutenin subunits (HMW-GSs and LMW-GSs), which constitute approximately 40% and 60% of the total glutenin content, respectively [6,7]. HMW-GSs are also responsible for almost 60% of the phenotypic differences in the baking quality of wheat flour and contribute significantly to dough strength and elasticity, which are essential for achieving optimal bread volume and texture [8–10]. In turn, monomeric gliadins, which constitute approximately 60% of the total gluten content, primarily contribute to the viscosity and extensibility dough and are less important for overall dough performance [11,12]. Gliadins are grouped into α/β-, γ- and ω-gliadins based on their decreasing electrophoretic mobility pattern at acidic pH [13–15] and serve as the primary environmental triggers of celiac disease (CD) [16–18]. Among the various gliadin subunits, α-gliadins contain the major toxic epitopes responsible for inducing CD in susceptible individuals [18,19].

In common wheat, HMW-GSs are encoded by *Glu-A1*, *Glu-B1* and *Glu-D1* on the long arms of group-1 chromosomes 1A, 1B and 1D, respectively [20]. Among these, *Glu-D1* locus has the most significant positive effect on dough quality [21]. Each *Glu* locus encodes two different x-type and y-type subunits including *Glu-1Ax*, *Glu-1Ay*, *Glu-1Bx*, *Glu-1By*, *Glu-1Dx*, and *Glu-1Dy*. Although up to six different subunits of HMW-GSs have been reported in wheat, many cultivars of common wheat only contain a combination of three to five HMW-GSs due to varying expression and silencing patterns of their associated genes [8]. For instance, to date, no commercial wheat variety has been found to contain an expressed 1Ay allele [22]. It has been mentioned that the presence of 1Dx5 + 1Dy10 (5 + 10) allelic combination is typically associated with excellent bread-making performance, whereas 1Dx2 + 1Dy12 (2 + 12) is generally linked to lower dough strength. Other subunits like 1Ax2* and 1Dx17 + 1Dy18 (17 + 18) have also been found to contribute positively to baking quality [23–27]. In general, correlations between HMW-GSs and dough quality have effectively been utilized to predict bread-making quality of wheat flour based on the *Glu-1* scoring system [6,8].

In recent decades, wheat breeding efforts have led to significant improvement in grain nutritional quality and various agronomic traits. For this, amphiploids and synthetic wheat lines involving wheat wild relatives provides an important source of the genetic diversity for the enrichment of the grain protein content [28–37]. We already developed several distinct synthetic and amphiploid wheat lines (Table 1) by cross-hybridizing various tetraploid wheat genotypes with different *Aegilops* species. These include emmer wheat (*T.dicoccum* or *T. dicoccoides*; AABB) × *Ae. tauschii* (2n = DD or DDDD), *T. durum* (AABB) × *Ae. tauschii*, *T. timopheevii* (A^t^A^t^GG) × *Ae. tauschii*, *Ae.* crassa (D^1^D^1^X^cr^X^cr^) × *T. durum*, and *Ae. ventricosa* (D^v^D^v^N^v^N^v^) × *T. durum* [38]. Here, we evaluated the gluten composition, grain protein content, and several quality-related gluten indices across 47 synthetic wheat lines or amphiploids resulted from these crosses. Additionally, we evaluated the HMW-GSs and compared with the previously characterized HMW-GSs associated with bread-making quality. We further identified synthetic lines possessing favorable glutenin subunits for bread processing that could be incorporated to enhance the nutritional quality and baking performance of modern wheat cultivars.

**Table 1:** Characterization of gliadin- and HMW-glutenin subunits (analyzed by Acid-PAGE/SDS-PAGE, respectively), allelic composition, grain protein content (%), wet gluten, and gluten index in 55 genotypes, including four common wheat cultivars, two durum wheat lines, and 49 synthetic wheat lines or amphiploids.

Lines	Genotypes	Genome	No. of gliadin bands			No. of HMW-GSs	HMW glutenin loci			Protein%	Wet Gluten	Gluten index
			ω	ϒ + β	α		Glu-A1	Glu- B1	Glu- D1			
**Line.01**	*T. aestivum* ‘Pishgam’	ABD	4	5	3	5	1	21 + 19	3 + 10	11.7	40	46
**Line.02**	*T. aestivum* ‘Chinese Spring’	ABD	6	8	4	4	Null	7 + 8	2 + 12	12.1	35	91
**Line.03**	*T. durum* ‘12595’ × Ae. tauschii ‘13939’	ABD	4	8	3	3	2*	7 + 8	Null	15.7	35	22
**Line.04**	*Ae. crassa* B × *T. durum* ‘6268’	ABD^1^X^cr^	3	6	2	4	Null	14 + 15	2 + 12.5	12.6	40	25
**Line.05**	*T. durum* ‘78’ × *Ae. tauschii* ‘1600’	ABD	6	5	3	4	Null	7 + 18	3 + 10	18	48	36
**Line.06**	*T. durum* ‘12595’ × *Ae. tauschii* ‘299’	ABD	4	6	2	4	2*	6 + 18	10.5	15.4	46	35
**Line.07**	*T. durum* ‘40’ × *Ae. tauschii* ‘299’	ABD	5	8	3	3	1	22	5	17.2	34	33
**Line.08**	*T. durum* ‘40’ × *Ae. crassa* ‘1873’	ABD^1^X^cr^	4	7	2	5	Null	21 + 22	3 + 5 + 10.5	18.2	54	42
**Line.09**	*T. dicoccum* ‘49663’ × *Ae. tauschii* ‘AE 1211’	ABD	4	8	3	3	Null	8 + 13	2	19.3	56	48
**Line.10**	*T. dicoccum* ‘TazeabadAliabad’ × *Ae. tauschii* ‘299’	ABD	5	10	3	4	2*	13 + 18	10	17.9	60	73
**Line.11**	(*Ae. ventricosa* ‘1496’ × *T. durum* ‘6268’) × *T. aestivum* ‘Bahmani’	ABD^v^N^v^	5	8	3	5	1	6 + 8	3 + 10.5	20.7	54	62
**Line.12**	*Ae. crassa* ‘1873’ × *T. durum* ‘40’	ABD^1^X^cr^	4	6	3	5	Null	14 + 18	2 + 4 + 12.5	20.2	NA	NA
**Line.13**	*T. dicoccum* ‘Tirgaran’ × *Ae. tauschii* ‘299’	ABD	5	8	4	4	Null	14 + 18	2 + 10.5	18.1	52	85
**Line.14**	*T. durum* ‘19850’ × *Ae. tauschii* ‘299’	ABD	4	9	3	4	2*	18	4 + 10.5	18.3	61	65
**Line.15**	*T. dicoccoides* ‘IG127697’ × *Ae. tauschii* ‘299’	ABD	6	7	2	3	2*	15	10.5	17.8	57	58
**Line.16**	*T.dicoccoides* ‘IG12638’ × *Ae. tauschii* ‘1211’	ABD	6	8	2	4	2*	6 + 19	10	19.9	62	51
**Line.17**	T*. durum* ‘78’ × *Ae. tauschii* ‘299’	ABD	5	8	3	5	1	14 + 15	5 + 10	19.4	45	49
**Line.18**	*T. timopheevii* × *Ae. tauschii* ‘1603’	A^t^GD	8	8	3	3	Null	21 + 15	3	18.4	NA	NA
**Line.19**	*T. timopheevii* × *Ae. tauschii* ‘143’	A^t^GD	3	7	2	3	2*	7 + 8 or 21 + 8	Null	23.5	76	46
**Line.20**	*T. durum* ‘78’ × *Ae. tauschii* ‘1211’	ABD	4	8	3	4	2*	6 + 15	10.5	16.7	48	69
**Line.21**	*T. timopheevii* × *Ae. Tauschii* ‘191’	A^t^GD	5	6	2	4	Null	21 + 15 + 9	3	22.7	72	24
**Line.22**	*T. timopheevii* × *Ae. tauschii* ‘1602’	A^t^GD	5	8	3	4	Null	21 + 19	3 + 10	21.2	68	26
**Line.23**	*T. durum* ‘12595’ × *Ae. tauschii* ‘13938’	ABD	3	9	3	3	1	21 + 18	Null	16	46	18
**Line.24**	*T.dicoccoides* ‘IG88732’ × *Ae. tauschii* ‘191’	ABD	3	9	2	3	Null	7 + 8	4	20.5	51	75
**Line.25**	(T. durum ‘40’ × Ae. tauschii ‘299’) × Pishgam	ABD	2	6	2	5	2*	21 + 17	4 + 10.5	13.2	NA	NA
**Line.26**	(*T. durum* ‘12595’ × *Ae. tauschii* ‘299’) × *T. aestivum* ‘Pishgam’	ABD	1	6	2	5	1	21 + 15	3 + 10.5	13	32	89
**Line.27**	(*T. durum* ‘19850’ × *Ae. crassa* ‘1874’) × *T. aestivum* ‘Bahmani’	ABD^1^X^cr^	3	8	2	6	Null	21 + 14 + 17 + 18	2 + 12	16.5	NA	NA
**Line.28**	(*T. durum* × *Ae. tauschii* ‘299’) × *T. aestivum* ‘Pishgam’	ABD	2	6	3	4	1	21	3 + 10	13	38	76
**Line.29**	(*T. durum* ‘78’ × *Ae. tauschii* ‘299’) × *T. aestivum* ‘Bahmani’	ABD	3	7	3	NA	NA	NA	NA	NA	NA	NA
**Line.30**	(*Ae. ventricosa* ‘1496’ × *T. durum* ‘6268’) × *T. aestivum* ‘Bahmani’	ABD^v^N^v^	4	7	3	NA	NA	NA	NA	NA	NA	NA
**Line.31**	(*Ae. ventricosa* ‘1522’ × *T. durum* ‘1’) × *T. aestivum* ‘Pishgam’	ABD^v^N^v^	4	7	2	5	1	13 + 19	3 + 4	17.1	55	56
**Line.32**	*T. dicoccum* ‘49666’ × *Ae. tauschii* ‘AE 1211’	ABD	5	7	3	5	1	13 + 16 + 9	3	20.5	63	43
**Line.33**	*Ae. crassa* B × *T. durum* ‘14’	ABD^1^X^cr^	4	6	3	4	Null	6 + 8	2 + 12.5	18.6	56	38
**Line.34**	*T. dicoccum* ‘Bainjub’ × *Ae. tauschii* ‘1211’	ABD	5	7	3	5	1	13 + 16 + 9	3	17.3	55	45
**Line.35**	*Ae. ventricosa* × *T. durum* ‘12089’	ABD^v^N^v^	7	6	3	5	1	7 + 17	3 + 4 + 12.5	22.5	62	51
**Line.36**	(*Ae. ventricosa* ‘1522’ × *T. durum* ‘8’) × *T. aestivum* ‘Pishgam’	ABD^v^N^v^	6	7	2	NA	NA	NA	NA	15.7	48	68
**Line.37**	*Ae. ventricosa* ‘1522’ × *T. durum* ‘13’	ABD^v^N^v^	5	6	3	4	Null	6 + 13	2.2 + 10	14.1	NA	NA
**Line.38**	(*T. durum* ‘78’ × *Ae. tauschii* ‘299’) × *T. aestivum* ‘Bahmani’	ABD	3	7	3	4	Null	14 + 9	4 + 12.5	16.8	49	85
**Line.39**	(*Ae. crassa* ‘1873’ × *T. durum* ‘40’) × *T. aestivum* ‘Pishgam’	ABD^1^X^cr^	6	9	2	6	1	13 + 19 + 9	3 + 5	NA	41	88
**Line.40**	(*T. durum* ‘10’ × *Ae. tauschii* ‘299’) × *T. aestivum* ‘Pishgam’	ABD	1	7	3	6	1	21 + 19	3 + 5 + 10.5	NA	NA	NA
**Line.41**	((*T. dicoccum* ‘Javanmard’ × *T. dicoccoides* ‘Seysaleh’) × *Ae. tauschii* ‘1211’) × *T. aestivum* ‘Pishgam’	ABD	1	6	2	4	Null	21 + 19	3 + 10	14.5	NA	NA
**Line.42**	*Ae. crassa* S × *T. durum* ‘6268’	ABD^1^X^cr^	4	7	2	3	Null	14	2 + 12.5	16.7	NA	NA
**Line.43**	*T.dicoccoides* ‘IG88753’ × *Ae. tauschii* ‘299’	ABD	4	8	3	3	2*	Null	5 + 10/10.5	NA	NA	NA
**Line.44**	(*T. durum* ‘19634’ × *Ae. tauschii* ‘36751’) × *T. aestivum* ‘Pishgam’	ABD	3	5	2	NA	NA	NA	NA	NA	NA	NA
**Line.60**	((*T. dicoccum* ‘Javanmard’ × *T. dicoccoides* ‘Seysaleh’) × *Ae. tauschii* ‘1211’) × *T. aestivum* ‘Pishgam’	ABD	4	8	3	NA	NA	NA	NA	NA	NA	NA
**Line.61**	(*T. durum* ‘40’ × *Ae. tauschii* ‘299’) × *T. aestivum* ‘Pishgam’	ABD	5	7	3	NA	NA	NA	NA	NA	NA	NA
**Line.78**	*T. dicoccoides* ‘Seysaleh’	AB	5	9	2	NA	NA	NA	NA	NA	NA	NA
**Line.79**	*T. dicoccum* ‘Javanmard’	AB	3	5	2	NA	NA	NA	NA	NA	NA	NA
**Line.82**	*T. dicoccoides* ‘Hawraman’ × *T. dicoccum* ‘Qara-Boghreh’	AB	NA	NA	NA	3	*	7 + 8	Null	NA	NA	NA
**Line.86**	*T. dicoccum* ‘Qara-Boghreh’ × *T. dicoccoides* ‘Seysaleh’	AB	NA	NA	NA	5	2.1	13 + 16 + 22	5	NA	NA	NA
**Line.87**	*T. aestivum* ‘Baran’	ABD	NA	NA	NA	5	1	6 + 22	5 + 10.5	NA	NA	NA
**Line.89**	*T. aestivum* ‘Roshan’	ABD	NA	NA	NA	4	Null	14 + 15	2 + 12.5	NA	NA	NA
**Line.90**	(*T. durum* ‘40’ × *Ae. tauschii* ‘299’) × *T. aestivum* ‘Pishgam’	ABD	5	7	3	5	2*	6 + 22	10.5	12.6	38	89
**Line.92**	*T. durum* ‘40’	AB	2	6	2	2	Null	21 + 8	Null	NA	NA	NA
**Line.93**	*T. durum* ‘87’	AB	NA	NA	NA	3	Null	6 + 20	Null	NA	NA	NA

NA (not available).

## Materials and Methods

### Plant materials

A collection of plant materials, including 4 common wheat cultivars, 2 durum wheat lines, and 49 synthetic wheat lines or amphiploids (Table 1) were cultivated during autumn 2022 in a field located at the University of Kurdistan, Iran. All genotypes were grown under uniform conditions using homogeneous soil and without the application of chemical fertilizers. Seeds were harvested in early summer 2023 and stored in paper bags under ambient conditions until protein extraction and further biochemical analyses.

### Acid-PAGE analysis of gliadin proteins

Gliadin proteins were first extracted from total wheat protein by adding 200 µl of 70% (v/v) ethanol to 30 mg of finely milled flour, following the method described by Khan et al. (1985) [39] with minor changes. To dissolve and extract gliadins in ethanol, the samples were vortexed for 30 seconds every 10 minutes over a total duration of 1 hour while kept on ice. Subsequently, the samples were centrifuged at 12,000 rpm for 15 minutes. The resulting supernatant was transferred to a new 1.5 mL microcentrifuge tube and used for analyzing the gliadin banding pattern via acid-polyacrylamide gel electrophoresis (Acid-PAGE). To perform Acid-PAGE, 20 µl of the gliadin-containing supernatant was incubated at 70 °C in a water bath for 5 minutes. The resulting pellet was dissolved in 40 µl of sample buffer consisting of 4.5 M urea prepared in 5% (v/v) glacial acetic acid. From each sample, 6 µl of the protein solution was loaded onto a 10% (w/v) acrylamide gel. Electrophoresis was carried out for 90 minutes at 400 V following the previous protocols [19,40]. Gel images were captured using a digital camera. Gliadin banding patterns for each genotype were compared with those of the reference cultivar Chinese Spring (CS) wheat and annotated accordingly.

### SDS-PAGE analysis of HMW-GSs

Glutenin proteins were analyzed via sodium dodecyl sulfate polyacrylamide gel electrophoresis (SDS-PAGE). For this, non-glutenin protein fractions were selectively removed from wheat flour [41]. Briefly, 30 mg of finely milled wheat flour was transferred into a 1.5 mL microtube. Sequential extractions were performed to eliminate non-glutenin fractions: albumins were removed using autoclaved distilled water, globulins with 0.5 M NaCl, and gliadins with 70% (v/v) ethanol. Each extraction step involved the addition of 300 µl of the respective extraction buffer to the flour, followed by incubation at 4 °C for 30 minutes. During incubation, samples were vortexed for 1 minute at 10-minutes intervals. The mixture was then centrifuged at 10,000 rpm for 3 minutes, and the supernatant containing the extracted non-glutenin fraction was discarded. After the sequential removal of albumins, globulins, and gliadins, glutenins were extracted by adding 300 µl of glutenin extraction buffer comprising 2% (w/v) sodium dodecyl sulfate (SDS), 6 M urea, and 1.5% (v/v) 2-mercaptoethanol to the remaining pellet. The mixture was vortexed for 1 minute every 10-minutes over 1 hour incubation period on ice, followed by centrifugation at 10,000 rpm for 10 minutes to collect the supernatant containing the solubilized glutenins. An aliquot of 40 µl of the glutenin-containing supernatant was mixed with an equal volume of sample buffer comprising 62.5 mM tris-HCl (pH 6.8), 10% (v/v) glycerol, 2% (w/v) SDS, 0.002% bromophenol blue and 5% (v/v) mercaptoethanol. The mixture was then stored at −20 °C until further analysis by SDS-PAGE. Gel preparation was performed according to the method described by Singh et al. (1991) [42]. The separating gel consisted of 0.36 M Tris-HCL (pH 8.8), 0.1% (w/v) SDS and 10% (w/v) acrylamide and 0.125% (w/v) N,N′-methylenebisacrylamide (Bis). The stacking gel contained 3% (w/v) acrylamide, 0.25% (w/v) Bis, 0.1% (w/v) SDS, and 0.006 M Tris-phosphate (pH 6.8). Prior to electrophoresis, protein samples were incubated at 70 °C for 15 minutes to facilitate denaturation, followed by vertexing for 10 seconds. Subsequently, 5 µl of each sample was loaded into the wells of the SDS-PAGE gel. Electrophoresis was performed at 80 V for 30 minutes to allow stacking, followed by 120 V for 4.5 hours. Gels were stained with 0.2% (w/v) Coomassie Brilliant Blue G-250 for 2–3 hours on a shaker, then briefly de-stained in distilled water for 10–20 minutes. Gel images were captured using a digital camera.

### Identification of the prolamin protein subunits

**Fig 1:**
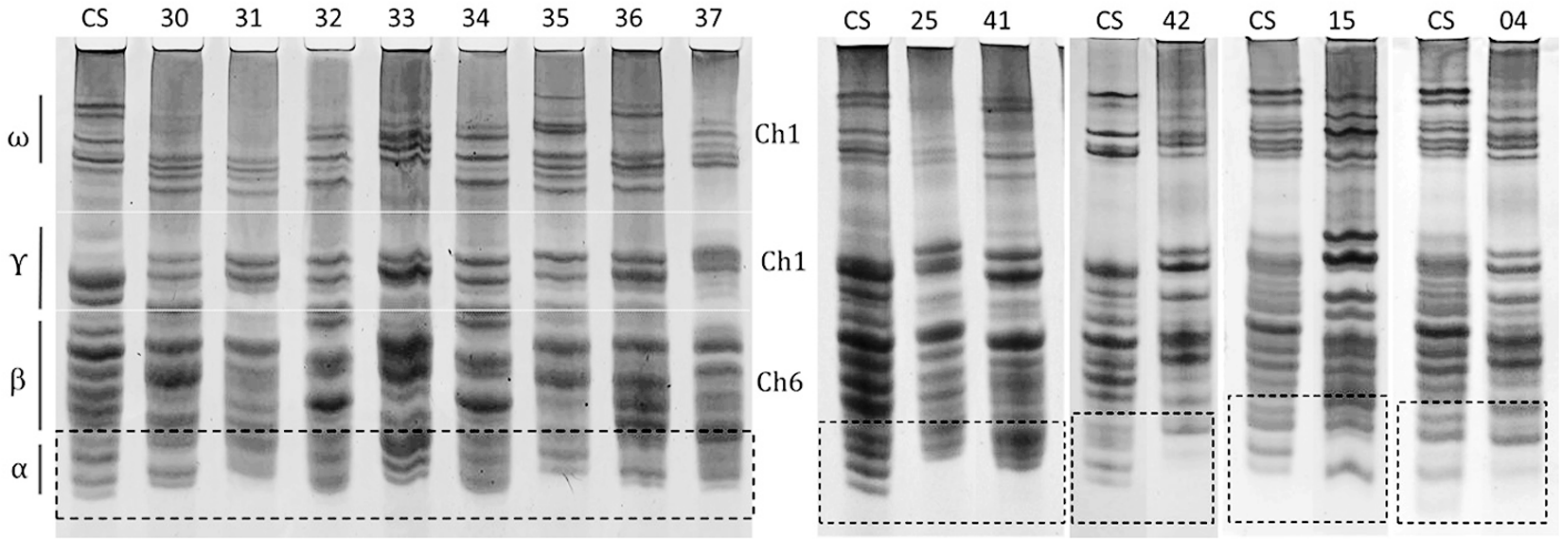
Acid-PAGE analysis of gliadin proteins in synthetic wheat lines (Table 1), compared to the reference cultivar Chinese Spring (CS), revealed distinct electrophoretic profiles. Gliadin subunits are indicated by vertical black lines, with their corresponding chromosomal locations (Ch1 and Ch6) annotated between the two Acid-PAGE gels. α-gliadin subunits from individual lines are highlighted within dashed rectangles.

Distinct gliadin subunits including α/β-, γ- and ω-gliadins were identified and classified based on their electrophoretic mobility and banding patterns relative to those of Chinese Spring wheat as described by Watry et al. (2020) [40]. HMW-GSs and LMW-GSs were distinguished using a protein marker ladder, as well as by referencing the positions of the HMW-GS bands in Chinese Spring wheat (Fig 1A and S2 Fig). The identification of HMW-GSs at *Glu-A1*, *Glu-B1*, and *Glu-Dt1* loci was carried out according to the classification and nomenclature systems of Payne and Lawrence, (1983), Pena et al. 1995, and William et al. 1993 [27,43,44]. These systems have been previously applied to Chinese Spring wheat, which carries the allelic combinations *Glu-A1* (Null), *Glu-B1* (7 + 8) and *Glu-D1* (2 + 12), as well as to a reference panel of 300 varieties of hexaploid wheat at the corresponding loci.

### Determination of grain protein content

The grain protein content of the synthetic wheat lines was determined using the Kjeldahl method [45]. Briefly, total nitrogen content was quantified from 0.5 g of wheat grain flour following standard digestion and distillation procedures. Crude protein content was then calculated using the wheat-specific nitrogen-to-protein conversion factor of 5.81 [46], according to the following equations:


$$\text{Nitrogen}\;\text{content}\;(\%)\;=\frac{\left(V_{\text{1}}-V_{\text{2}}\right)\times N_{HCl}\;\times \text{14}.\text{007}}{W_{sample}\times \text{10}}$$

Crude protein (%) = Nitrogen (%) × 5.81

Where: V1 = Volume of HCl consumed for the sample (mL), V2 = Volume of HCl consumed for the blank (mL), N = Normality of HCl (equiv./L), W = Weight of the sample (g), 14.007 = Atomic weight of nitrogen (g/mol).

### Gluten content and strength evaluation

Wet gluten was measured following the methods described by Perten (1990) [47] and Mcdonald (1994) [48]. Briefly, 10 g of wheat flour was mixed with 5 mL of distilled water to form a dough, which was then washed under running water for 5–10 minutes to remove water-soluble components. The remaining gluten mass was collected and weighed, and the wet gluten content (%) was calculated as the weight of the wet gluten relative to the original flour weight. To assess gluten strength, the wet gluten was placed in a mesh sieve and centrifuged at 6,000 rpm for 1 minute. The gluten index was calculated as the ratio of the gluten retained on the sieve after centrifugation to the total wet gluten mass. A gluten index above 75% typically indicates strong gluten, while values bellow 50% indicate weak gluten.

### Variation analysis based on prolamin banding patterns

Protein bands from Acid-PAGE and SDS-PAGE gels were scored using a binary system, with the presence of a band assigned a value of 1 and its absence assigned 0 for each allele. A clustering dendrogram was generated based on these binary scores for gliadin and glutenin banding patterns using NAM [49] and dendextend [50] packages in R.

## Results

### Characterization and distribution of gliadin subunits

Gliadin banding patterns of 50 genotypes including 45 synthetic or amphiploid wheat lines, one durum wheat, two common wheat cultivars, and two emmer lines (Table 1) were analyzed using Acid-PAGE. A total of 696 gliadin bands were detected across all genotypes. Among these, 51% corresponded to γ- and β-gliadins, 30% to ω-gliadins, and the remaining 19% were α-gliadins. The total number of gliadin bands per genotype varied between 9 and 19 bands. A total of 49 distinct electrophoretic gliadin banding patterns were identified in the studied genotypes in comparison to Chinese Spring wheat, indicating that each line exhibited a unique gliadin profile. (S1 Table, Fig 1 and S1 Fig). The lowest numbers of gliadin bands (9–11) were primarily observed in backcrossed lines ((*T. durum* × *Ae. tauschii*) × *T. aestivum* ‘Pishgam’). In contrast, the highest numbers of bands (17–19) were detected in approximately 10% of the genotypes, predominantly in amphiploid lines derived from *T. dicoccum* × *Ae. tauschii* ‘299’ and *T. timopheevii* × *Ae. tauschii* ‘1603’.

Further analysis of gliadins revealed distinct distributions of individual subunit types among the lines (S2 Table). For instance, ω-gliadins ranged from 1 to 8 bands, with 54% of the lines exhibiting 4–5 bands. γ- and β-gliadins showed a broader range of 5–10 bands, with 6–8 bands prevalent in 80% of the lines. In contrast, α-gliadins showed 1–4 bands, with 2 or 3 subunits being the most common, occurring in 38% and 54% of the lines, respectively.

About 25% of the synthetic lines primarily derived from crosses between *T. dicoccoides* × *Ae. tauschii* and *Ae. ventricosa* × *T. durum*, exhibited higher numbers of ω-gliadin subunits (6–7 bands). 35% of the synthetic lines derived from *T. durum* × *Ae. tauschii* and 40% which derived from emmer wheat × *Ae. tauschii* showed elevated number of γ- and β-gliadin bands (8–10 bands). Similarly, lines with a higher number of α-gliadin subunits (3 bands) were mainly the result of crosses involving *T. durum* × *Ae. tauschii* (46%), emmer × *Ae. tauschii* (18%), and *Ae. ventricosa* × *T. durum* (14%). In contrast, lines with fewer α-gliadin subunits (2 bands) were found among genotypes with varied genome compositions, with the highest frequencies found in those derived from combinations of durum wheat × *Ae. crassa*, emmer × *Ae. tauschii*, and durum wheat × *Ae. tauschii* (S2 Table).

### Characterization and distribution of HMW-GSs

**Fig 2:**
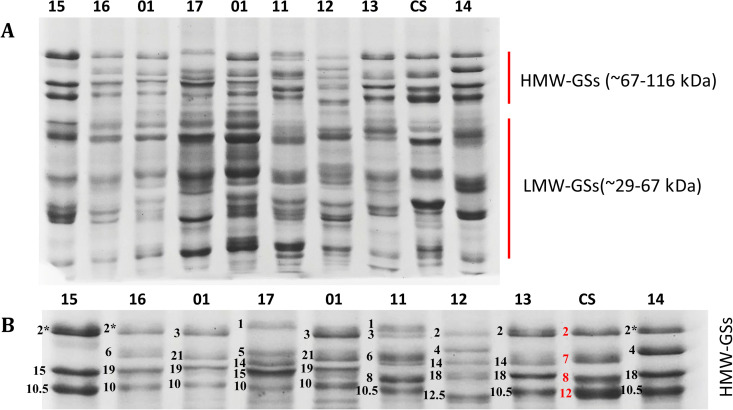
(A) SDS-PAGE profiles and molecular weight analysis of high- and low-molecular-weight glutenin subunits (HMW-GSs and LMW-GSs) in synthetic wheat lines (Table 1), compared to the reference cultivar Chinese Spring (CS). (B) Classification and variation of HMW-GSs among the synthetic wheat lines.

Forty-one synthetic/amphiploid wheat lines, 2 durum wheat lines, and 4 common wheat cultivars were analyzed for their HMW-GSs composition and frequency. The HMW-glutenin alleles were classified and named according to the scoring system of Payne and Lawrence, (1983) [27]. A total of 198 HMW-glutenin bands and 47 distinct patterns of HMW-GSs were identified across all studied lines (Table 1 and S3 Table, Fig 2B and S3 Fig). Two to six different HMW-GSs were detected across these lines. Notably, 38.3% (18 out of 47 lines) of the lines contained four HMW-GSs, primarily from *T. durum* × *Ae. tauschii* and emmer × *Ae. tauschii*. Additionally, 25.5% of the lines had three HMW-GSs, mainly from emmer × *Ae. tauschii*, while 29.8% of the crosses exhibited five HMW-GSs, predominantly from *T. durum* × *Ae. tauschii*. Lines with two HMW-GSs were the least frequent, comprising only 2.1% of the total. The lines with higher number of HMW-GSs (six) originated from the amphiploids *T. durum* × *Ae. crassa*.

Four distinct HMW-GSs (Null, 1, 2 and 2*) were identified at the *Glu-A1* locus (Table 1). The Null, 1, and 2* alleles were the predominant subunits, which accounted for approximately 42.5%, 30%, and 23.5% of the synthetic lines, respectively. Lines exhibiting the Null allele were predominantly derived from the crosses of *Ae. crassa* × *T. durum*, *T. timopheevii* × *Ae. tauschii*, and emmer × *Ae. tauschii*. In contrast, lines with the HMW-glutenin subunit 1 primarily originated from the *T. durum* × *Ae. tauschii* crosses, whereas those with the 2* allele were sourced from both *T. durum* × *Ae. tauschii* and emmer × *Ae. tauschii*.

At the *Glu-B1* locus, we detected 34 distinct HMW-GS allelic patterns (Table 1). The most frequent subunits at this locus were 21 + 19, 7 + 8, 14 + 15, 6 + 8, 14 + 18, 21 + 15, 13 + 16 + 9, and 6 + 22, which collectively accounted for 51% of the lines. These patterns were primarily found in the crosses of *T. durum* × *Ae. tauschii*, *T. timopheevii* × *Ae. tauschii*, emmer × *Ae. tauschii*, and *Ae. crassa* × *T. durum*. Interestingly, Line 21 (an amphiploid from *T. timopheevii* × *Ae. Tauschii* with A^t^GD genome), line 32 and 34 (*T. dicoccum* × *Ae. tauschii* amphiploids), line 39 ((*Ae. crassa* ‘1873’ × *T. durum* ‘40’) × *T. aestivum* ‘Pishgam’) and line 86 (an amphiploid from *T. dicoccum* × *T. dicoccoides*) showed three, and the line (*T. durum* ‘19850’ × *Ae. crassa* ‘1874’) × *T. aestivum* ‘Bahmani’ showed four HMW-GSs at *Glu-B1* locus.

At the *Glu-D1* locus, 22 distinct HMW-GS allelic patterns were detected (Table 1). The most prevalent patterns included alleles 3 + 10 or 3 + 10.5, which comprised 19% of the lines, primarily from *T. durum* × *Ae. tauschii*. Additionally, subunits 2 + 12 or 2 + 12.5 accounted for 15% of the lines and were mainly derived from *Ae. crassa* × *T. durum*, and subunits 5 + 10 or 5 + 10.5 from *T. durum* × *Ae. tauschii*. Remarkably, two *T. durum* × *Ae. crassa* (ABD^1^X^cr^) amphiploids (lines 08, 12), an *Ae. ventricosa* × *T. durum* (ABD^v^N^v^) amphiploid and a synthetic wheat derived line (line 40) exhibited three HMW-GSs at *Glu-D1* locus (Table 1).

### Grain protein content and gluten strength

A total of 38 wheat genotypes, including 36 synthetic or amphiploid lines and two common wheat cultivars, were evaluated for grain protein content. Additionally, a subset of 33 genotypes, comprising 31 synthetic or amphiploid lines and the same two common wheat cultivars was further assessed for additional quality parameters, including wet gluten content, gluten index, moisture, ash, and Zeleny sedimentation (Table 1 and S4 Table).

The synthetic wheat lines were classified into three groups based on their protein content (Table 1): low (<15%), medium (15–18%), and high (>18%). The low-protein group comprised 21% of the lines, predominantly derived from the backcrosses between *T. durum* × *Ae. tauschii* ‘299’ and *T. aestivum* ‘Pishgam’. The medium-protein group accounted for 34% of the lines, mostly originating from *T. durum* × *Ae. tauschii* crosses. The high-protein group represented 45% of the lines, primarily consisting of progeny from hybrids of *T. dicoccum*/*dicoccoides* × *Ae. tauschii* and *T. timopheevii* × *Ae. tauschii*.

The synthetic wheat lines were further categorized into three groups based on their gluten index (GI) (Table 1): weak (<30%), normal (30–80%), and strong (>80%) [51]. Fifteen percent of lines belonged to the weak-GI group, primarily consisting of progeny from *T. durum* ‘12595’ × *Ae. tauschii* ‘13939’ and *T. timopheevii* × *Ae. tauschii* ‘191’. The majority (67%) exhibited normal gluten strength, predominantly derived from crosses involving *T. durum* × *Ae. tauschii*, *Ae. ventricosa* × *T. durum*, and *T. dicoccum*/*dicoccoides* × *Ae. tauschii*. Only 18% of lines demonstrated strong gluten properties, largely represented by backcross lines from (*T. durum* × *Ae. tauschii*) × common wheat crosses.

In summary, 53% of the lines exhibited HMW-GSs at one or two *Glu* loci associated with favorable bread-making properties. These lines generally demonstrated a normal gluten index, except for lines 03 and 13 (Table 1 and S5 Table). Among these, 80% were primarily derived from crosses between *T. durum* × *Ae. tauschii*, and emmer wheat × *Ae. tauschii*. Additionally, correlations among grain protein content, wet gluten, gluten index, and Zeleny sedimentation were evaluated in these lines. Protein content showed a strong positive correlation with both Zeleny sedimentation (r = 0.895, P-value = 0.001) and wet gluten content (r = 0.824, P-value = 0.001). Furthermore, wet gluten was positively correlated with Zeleny sedimentation (r = 0.714, P-value = 0.001). No other significant positive or negative correlations were observed among the measured parameters (S4 Fig).

### Clustering analysis of synthetic wheats

**Fig 3:**
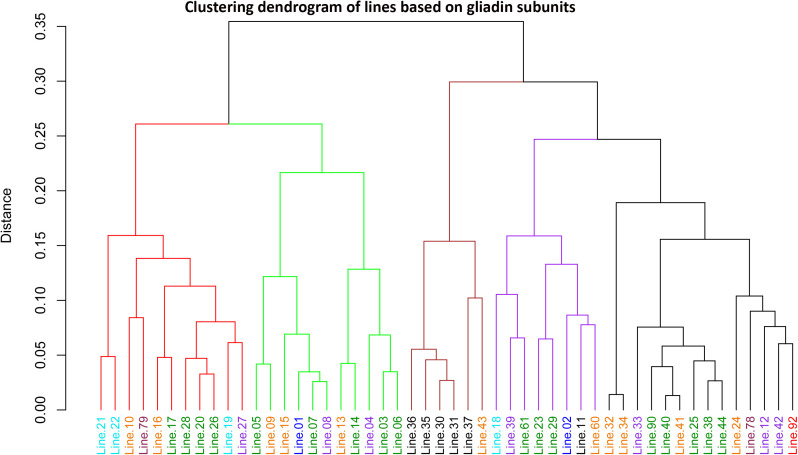
Clustering dendrogram of synthetic and amphiploid wheat lines based on Acid-PAGE banding pattens of gliadin protein subunits. Lines highlighted in the same color represent similar genomic compositions, as detailed in Table 1.

**Fig 4:**
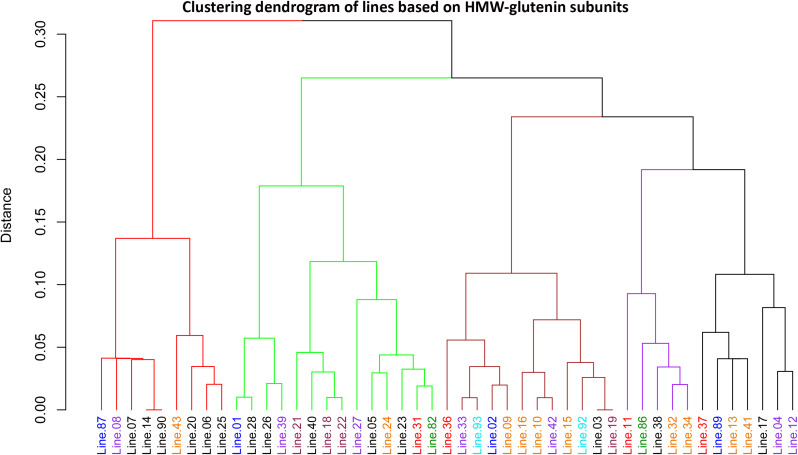
Clustering dendrogram of synthetic and amphiploid wheat lines based on SDS-PAGE banding patterns of high molecular weight glutenin subunits (HMW-GSs). Lines highlighted in the same color represent similar genomic compositions, as detailed in Table 1.

Clustering analysis based on the binary scores of gliadin and HMW-GS banding patterns (Figs 1 and 2, S1, S3 Figs) revealed that the synthetic wheat lines grouped into five distinct clusters (Figs 3 and 4). In both dendrograms, lines were color-coded according to their genomic compositions (Table 1). In the dendrogram constructed from gliadins profiles, amphiploids derived from *Ae. ventricosa* × *T. durum* (lines 30, 31, 35, 36, and 37) clustered together within the third group (Fig 3, indicated in black), whereas other lines were more dispersed and did not cluster strictly according to the genomic background. Although, some lines with similar genomic origins tended to co-cluster, the clustering based on HMW-GSs patterns did not correspond well to the genomic composition of the studied lines (Fig 4).

## Discussion

Common wheat varieties typically contains moderate protein levels (8–15%) [1]. Therefore, the development of novel and synthetic wheat lines with enhanced protein content while retaining desirable bread-making quality, is critical. In the present study, we evaluated 47 synthetic wheat lines or amphiploids resulted from the crosses between emmer wheat, durum wheat, *T. timopheevii*, *Ae. crassa*, *Ae. ventricosa* and *Ae. tauschii* (Table 1) for their glutenin and gliadin composition, protein content, wet gluten content, and some quality-related gluten indices.

### Gliadin subunits in synthetic wheat lines

A high variation in the composition of gliadin subunits was observed, even among synthetic lines with similar genomic backgrounds. This variation may be attributed, at least in part, to genetic differences among the parental genotypes used in hybrids formation. As a result, all 50 genotypes evaluated for their gliadin subunits composition exhibited distinct electrophoretic banding patterns. Among gliadin proteins, α-gliadins are recognized as the most immunogenic subunits involved in celiac disease (CD), an autoimmune disorder triggered by the consumption of wheat gluten [52]. Previous studies have demonstrated that reducing α-gliadin levels can effectively decrease the immunogenicity of wheat gluten, thereby contributing to improved health outcomes in individuals susceptible to CD [53,54]. In the present study, approximately one third of the lines (34%) exhibited a reduced number of α-gliadin bands, the majority of which were derived from *Ae. crassa*. Moreover, a positive correlation between the genome composition of synthetic wheat lines and their relative toxicity associated with CD has been previously reported [19]. In that study, synthetic lines derived from *Ae. crassa* showed significantly lower immunogenic potential compared to those derived from *Ae. tauschii* and other *Aegilops* species. Thus, identifying lines with fewer α-gliadin bands in our population highlights the substantial potential of D-genome-containing wild relatives, particularly *Ae. crassa*, for developing wheat varieties with reduced immunogenicity for individuals genetically susceptible to CD.

### HMW-GSs and their association with baking quality in synthetic wheat lines

The analysis of HMW-GSs in our lines revealed that the highest frequency of lines with four or more HMW-GS bands (over 72%) was found in the cross combinations of emmer wheat × *Ae. tauschii*, and durum wheat × *Ae. tauschii*. Similarly, the electrophoregram of HMW-GSs in ten different genotypes of emmer wheat and twenty-three hexaploid wheat lines (derived from bread wheat and wild emmer), predominantly displayed four to five distinct bands [55,56]. This suggests that various subunits of HMW-glutenins from synthetic wheat, which are derived from the AB sub-genome of emmer wheat and D sub-genome of *Ae. tauschii*, are actively expressed in the subsequent generations following cross-hybridization.

HMW-GSs play a critical role in determining the quality and bread-making properties of wheat flour. Developing synthetic wheat lines that incorporate a wide range of HMW-GSs offers breeders valuable genetic resources for improving wheat quality traits [57,58]. In particular, identifying and utilizing superior HMW-GSs from wheat wild relatives can significantly expand the genetic diversity of common wheat and enhance both its flour quality and nutritional value [59]. In terms of HMW-GSs at various Glu loci, we found that the most frequent subunits at the *Glu-A1* locus across our lines were Null, 1, and 2*. Similar allele distributions have been observed in previous studies. For example, a survey of 116 Spanish durum landraces reported frequencies of 51.7% for the Null allele and 23.3% for 2* [60]. Additionally, several studies on Mediterranean wheat landraces have shown that the frequency of the Null allele often exceeds 50% [61–64]. The high prevalence of the Null and 2* subunits in our lines likely reflects the genetic contribution of the different tetraploid wheat species used in our cross combinations. Li et al. (2019) [65] found that lines carrying subunit 1 exhibited better bread-making quality than those with subunit 2* or the Null allele. Other studies have shown that although subunits 1 and 2* are generally linked to improved bread-making quality, the Null allele can also enhance flour quality under certain conditions [5,7,66].

We also found that the diversity of HMW-glutenin alleles at the *Glu-B1* locus was considerably greater than that at other loci. Notably, 34 out of 60 observed allelic patterns were associated with the *Glu-B1*. This high level of diversity is believed to result from genetic polymorphism and allelic variation introduced by wheat relatives. Moreover, this diversity is strongly associated with improved baking quality in wheat [10,67]. With regard to different HMW-glutenin alleles at this locus, several studies have reported that the presence of the 21 + 19 and 13 + 16 alleles is associated with reduced wheat flour quality in certain regions [9,68]. In our study, these alleles were found in lines derived from emmer × *Ae. tauschii* crosses. Although some emmer cultivars demonstrate better baking quality than certain modern wheat varieties, they generally exhibit lower gluten index and Zeleny sedimentation values, traits we also observed in our lines. These characteristics make them less suitable for bread-making applications [69,70]. The subunit combinations 7 + 8 and 14 + 15, which have been reported to improve dough quality parameters, were detected in synthetic lines originating from various genomic backgrounds. Previous studies have also documented the presence of these subunits in both landraces and modern commercial cultivars [68,71]. Other studies have also linked the 6 + 8 subunit from synthetic wheat to superior overall quality characteristics compared to 7 + 8 [72]. In our research, this subunit was identified in lines derived from *Ae. ventricosa* or *Ae. crassa* × *T. durum* crosses. The 6 + 22 allele was detected in line derived from crosses between durum wheat and *Ae. tauschii*. Rai et al. (2018) [73] demonstrated that the presence of this allele at *Glu-B1* locus is vital for dough processing and bread-making, as it enhances the strength and viscoelasticity of wheat flour.

Variations in alleles at the *Glu-D1* locus play a significant role in determining dough quality [74]. At this locus, among 22 distinct allelic patterns, alleles 3 + 10 or 3 + 10.5, 2 + 12 or 2 + 12.5, and 5 + 10 or 5 + 10.5 were predominantly found in synthetic lines derived from crosses between durum wheat and *Ae. tauschii*. The presence of subunits combination of 5 + 10 is positively associated with dough strength, since subunit 5 contains an additional cysteine residue at the beginning of the repetitive domain, which make the flour more favorable for dough processing [8,75]. Moreover, Gupta et al. (1994) [76], found that the *Glu-D1d* allele (5 + 10) is linked to enhanced bread-making performance, whereas the *Glu-D1a* allele (2 + 12) is associated with reduced bread-making quality.

Although observing more than two HMW-GSs at each Glu locus is not common among bread wheat genotypes [8], we identified several lines expressing additional HMW-GSs at either *Glu-B1* or *Glu-D1* loci in lines 08, 12, 21, 27, 32, 34, 35, 39, 40, and 86 (see Table 1). The presence of additional bands could be due to the inclusion of subgenomes unusual in common wheat such as G from *T. timopheevii* (line 21), B subgenome from *T. dicoccum* or *T. dicoccoides* (e.g., lines 32 and 34); D^1^, X^cr^, D^v^ and N^v^ subgenomes from *Ae. crassa* (lines 08 and 12) or *Ae. ventricosa* (line 35), a combination of B and X^cr^ (in lines 27 and 39), heterozygosity for D subgenomes from common wheat and *Ae. tauschii* (in line 40) and heterozygosity for B subgenomes from *T. dicoccum* and *T. dicoccoides* (in line 86). Surprisingly, these lines (except line 40) have subgenomes in their pedigree which are unusual for the bread wheat (i.e., G, D^1^, X^cr^, D^v^, N^v^ and emmer wheat B subgenomes) (Table 1), suggesting the potential of the wheat wild relatives in enhancing the seed storage proteins of the common wheat.

### Protein content and gluten strength in synthetic lines

Bread wheat typically contains low protein content, rarely exceeding 15% [1]. However, in our analysis, 79% of the evaluated lines exhibited protein contents greater than 15%. All synthetic lines with elevated protein content had emmer, durum wheat, or *T. timopheevii* as one of the parental genotypes in their pedigree. The qualitative and quantitative composition of gluten proteins is determined by underlying genetic factors. In wild relatives and related species of wheat, this variability is influenced not only by differences in the genetic backgrounds but also by variations in nucleotide indels, different mutations, and differences in gene expression levels [77]. Numerous studies have demonstrated that the incorporation of wild relatives into wheat breeding programs can substantially enhance the protein content of bread wheat. For instance, Geisslitz et al. (2018) [78] analyzed crude protein contents in common wheat, spelt, durum wheat, and emmer, reporting significantly higher protein content in durum wheat compared to common wheat. Similarly, a comparative analysis of ancient and modern wheat species from various geographical locations revealed that both emmer and durum wheat contained higher protein content than hexaploid common wheat [79]. Additional studies have indicated that the introgression of genes from wild relatives into common wheat during its evolution has significantly contributed to enhancement of grain protein content. For example, a quantitative trait locus (*GPC-B1*), located on the short arm of chromosome 6B in *T. dicoccoides*, has various effects on grain protein content and is directly associated with the accumulation of protein in wheat grain [80,81]. Moreover, the *NAM-G1* gene from *T. timopheevii* and the *NAM-B1* gene from *T. dicoccoides* have been shown to enhance grain protein content and are recognized as key candidate genes for micronutrient enrichment in modern wheat cultivars [34,82,83]. Furthermore, Fatiukha et al. (2020) [84] identified quantitative trait nucleotides (*QTNs*) in wild emmer (*T. dicoccoides*) that are associated with increased protein levels. These genes can be successfully introgressed into modern wheat cultivars to improve both their nutritional profile and agronomic performance.

### Clustering analysis

In the dendrogram generated from gliadin subunits profile (Fig 3), lines with identical genome compositions were often clustered together. However, some divergence was observed, as lines with similar genomic backgrounds occasionally appeared in separate clusters. This variation is likely attributable to diverse parental genotypes used in the development of synthetic wheat lines, leading to differences in gliadin composition even among the genotypes with the same genome constitution. Similarly, Al-Khayri et al. (2023) [85] reported substantial variation in the electrophoretic banding patterns of gliadins, both among and within the various genotypes of durum and bread wheat. In contrast, clustering based on HMW-GSs rarely grouped lines with identical genome compositions, instead displaying a more dispersed pattern across genotypes compared to gliadin-based clustering (Fig 4). Although several lines exhibited the same number of HMW-GS bands, their electrophoretic allelic patterns differed significantly among the studied genotypes. In relation to observed variations in gliadin and glutenin composition, numerous studies have shown that genetic background, environmental factors, and their interactions play a significant role in determining both the content and relative proportions of gliadin and glutenin subunits in wheat [1,77,86].

## Conclusion

In this study, we conducted a comprehensive analysis of gluten subunit composition, grain protein content and key gluten-related quality parameters in a panel of synthetic wheat lines and amphiploids. The results revealed high variation in the gluten subunit profiles, total protein content and gluten strength among the genotypes. Notably, approximately 80% of the lines exhibited higher protein levels compared to common wheat. Several lines were identified as promising candidates for the development of wheat varieties with reduced CD-immunogenicity potential. In addition, specific lines carrying favorable HMW-glutenin alleles associated with enhanced baking quality were also identified. Together, these lines represent valuable genetic resources for the improvement of bread wheat with enhanced nutritional and functional properties.

## Supporting information

S1 Table Binary scoring and characterization of distinct gliadin subunit patterns in synthetic wheat lines and amphiploids, as determined by Acid-PAGE analysis. (XLSX)

S2 Table Frequency distribution of gliadin and glutenin protein subunits among synthetic wheat lines. (XLSX)

S3 Table Binary scoring and unique electrophoretic patterns of high molecular weight glutenin subunits (HMW-GSs) in synthetic wheat lines and amphiploids. (XLSX)

S4 Table Analysis of key flour quality parameters including protein content, wet gluten, moisture, ash, and Zeleny sedimentation values in synthetic wheat lines. (XLSX)

S5 Table HMW-glutenin subunits associated with bread-making quality of wheat in synthetic wheat lines. (XLSX)

S1 Fig Acid-PAGE banding patterns of gliadin subunits across the evaluated genotypes. The numbers above each lane correspond to different wheat genotypes, including common wheat, durum wheat, emmer lines, and synthetic wheat lines or amphiploids (Table 1).
(TIF)

S2 Fig SDS-PAGE banding patterns of HMW- and LMW-glutenin subunits in a representative subset of the synthetic lines. Lanes 1–9 correspond to the following genotypes: *T. aestivum* ‘Pishgam’, *T. aestivum* ‘Chinese Spring’, *T. durum* ‘12595’ × *Ae. tauschii* ‘13939’, *Ae. crassa* ‘B’ × *T. durum* ‘6268’, *T. durum* ‘78’ × *Ae. tauschii* ‘1600’, *T. durum* ‘12595’ × *Ae. tauschii* ‘299’, *T. durum* ‘40’ × *Ae. tauschii* ‘299’, *T. durum* ‘40’ × *Ae. crassa* ‘1873’, and *T. dicoccum* ‘49663’ × *Ae. tauschii* ‘AE 1211’, respectively.
(TIF)

S3 Fig SDS-PAGE banding patterns and classification of HMW-GSs across all genotypes. The numbers above each lane correspond to different wheat genotypes, including common wheat, durum wheat, emmer lines, and synthetic wheat lines or amphiploids (Table 1).
(TIF)

S4 Fig Correlation analysis of key wheat flour parameters, including protein content, wet gluten content, gluten index and Zeleny sedimentation value in synthetic wheat lines. (TIF)

## Abbreviations

HMW-GSs: High-molecular-weight glutenin subunits
SDS-PAGE: Sodium dodecyl sulfate-polyacrylamide gel electrophoresis
LMW-GSs: Low-molecular-weight glutenin subunits
Acid-PAGE: Acid-polyacrylamide gel electrophoresis
GPC: Grain protein content
WGC: Wet gluten content
CD: Celiac disease
rpm: Revolutions Per Minute
