# Supplementary figures and images for: High variation in the gluten composition and grain protein content among synthetic wheat lines
Source: PLoS One. 2025 Oct 10;20(10):e0331619. doi: 10.1371/journal.pone.0331619 (PMC12513617; doi:10.1371/journal.pone.0331619)

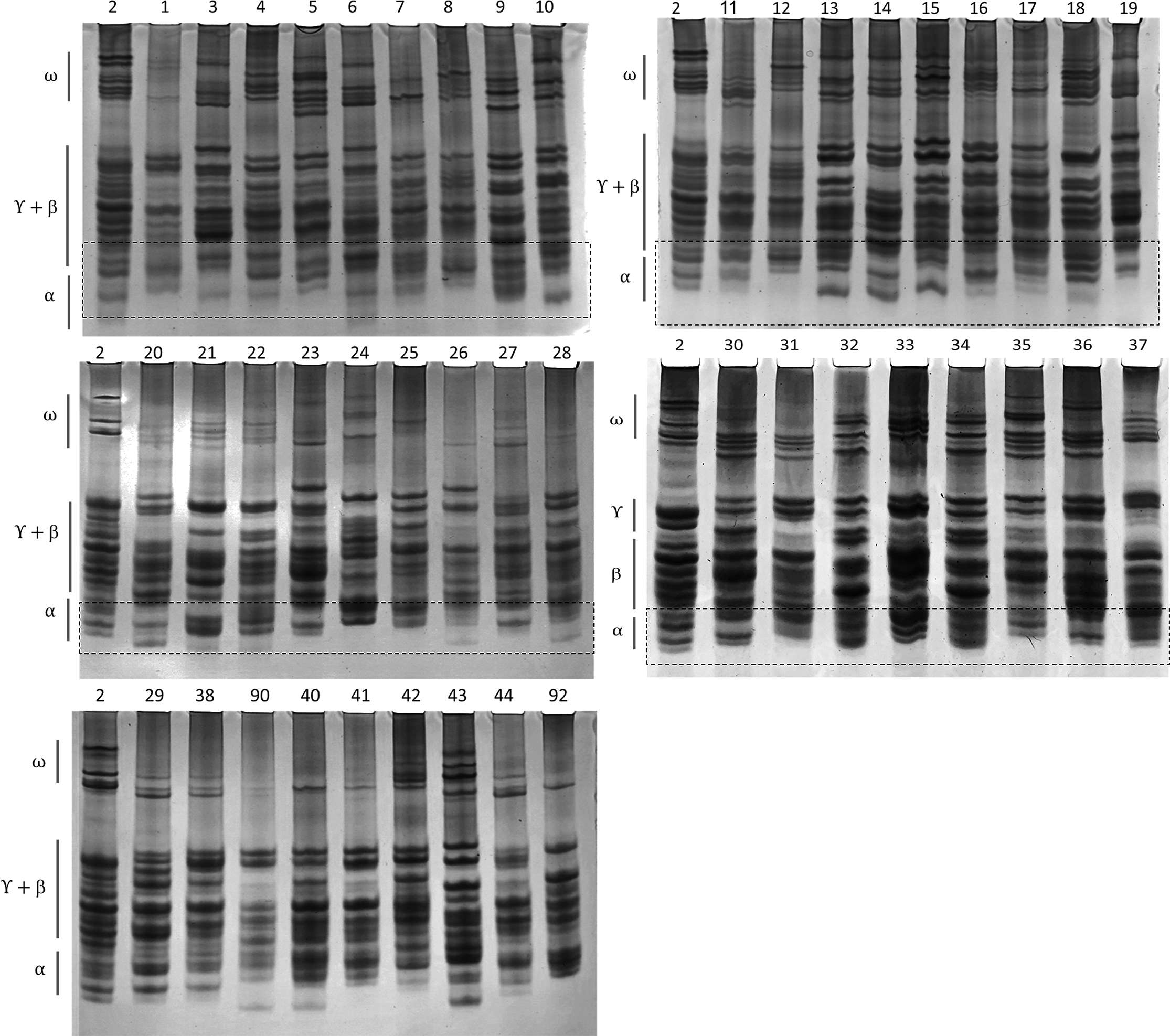

Supplement: S1 Fig — The numbers above each lane correspond to different wheat genotypes, including common wheat, durum wheat, emmer lines, and synthetic wheat lines or amphiploids (Table 1). (TIF) [file pone.0331619.s006.tif]

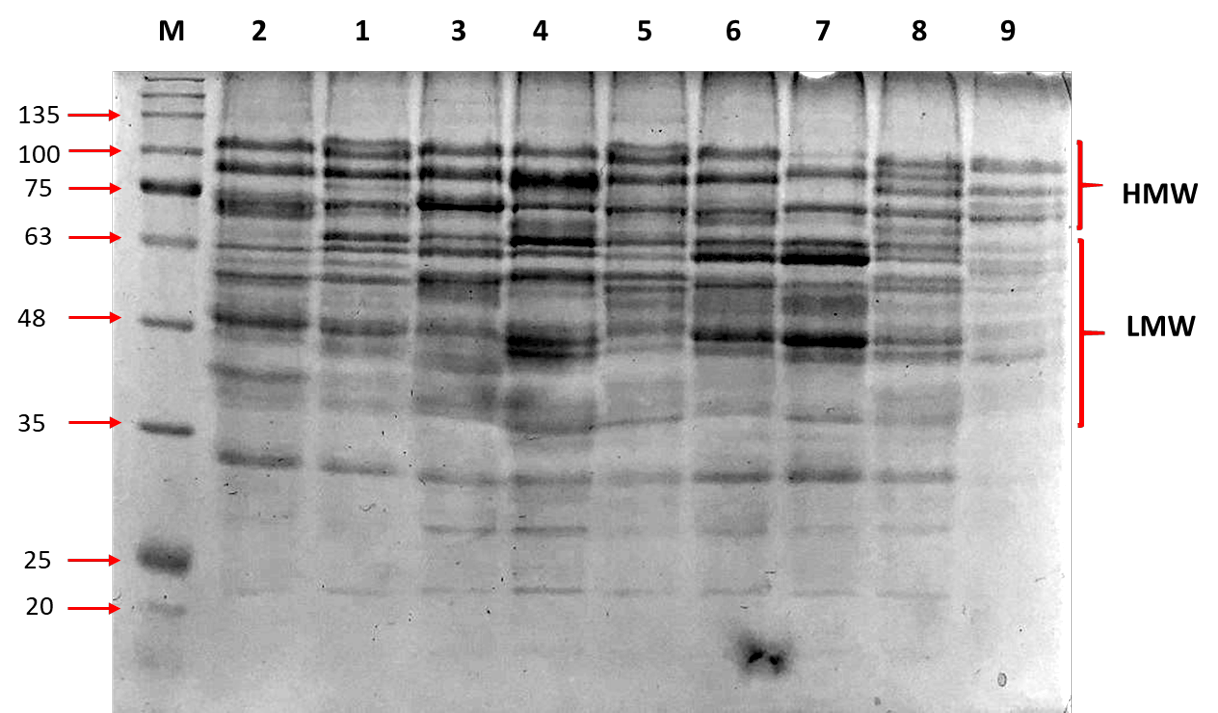

Supplement: S2 Fig — Lanes 1–9 correspond to the following genotypes: T. aestivum ‘Pishgam’, T. aestivum ‘Chinese Spring’, T. durum ‘12595’ × Ae. tauschii ‘13939’, Ae. crassa ‘B’ × T. durum ‘6268’, T. durum ‘78’ × Ae. tauschii ‘1600’, T. durum ‘12595’ × Ae. tauschii ‘299’, T. durum ‘40’ × Ae. tauschii ‘299’, T. durum ‘40’ × Ae. crassa ‘1873’, and T. dicoccum ‘49663’ × Ae. tauschii ‘AE 1211’, respectively. (TIF) [file pone.0331619.s007.tif]

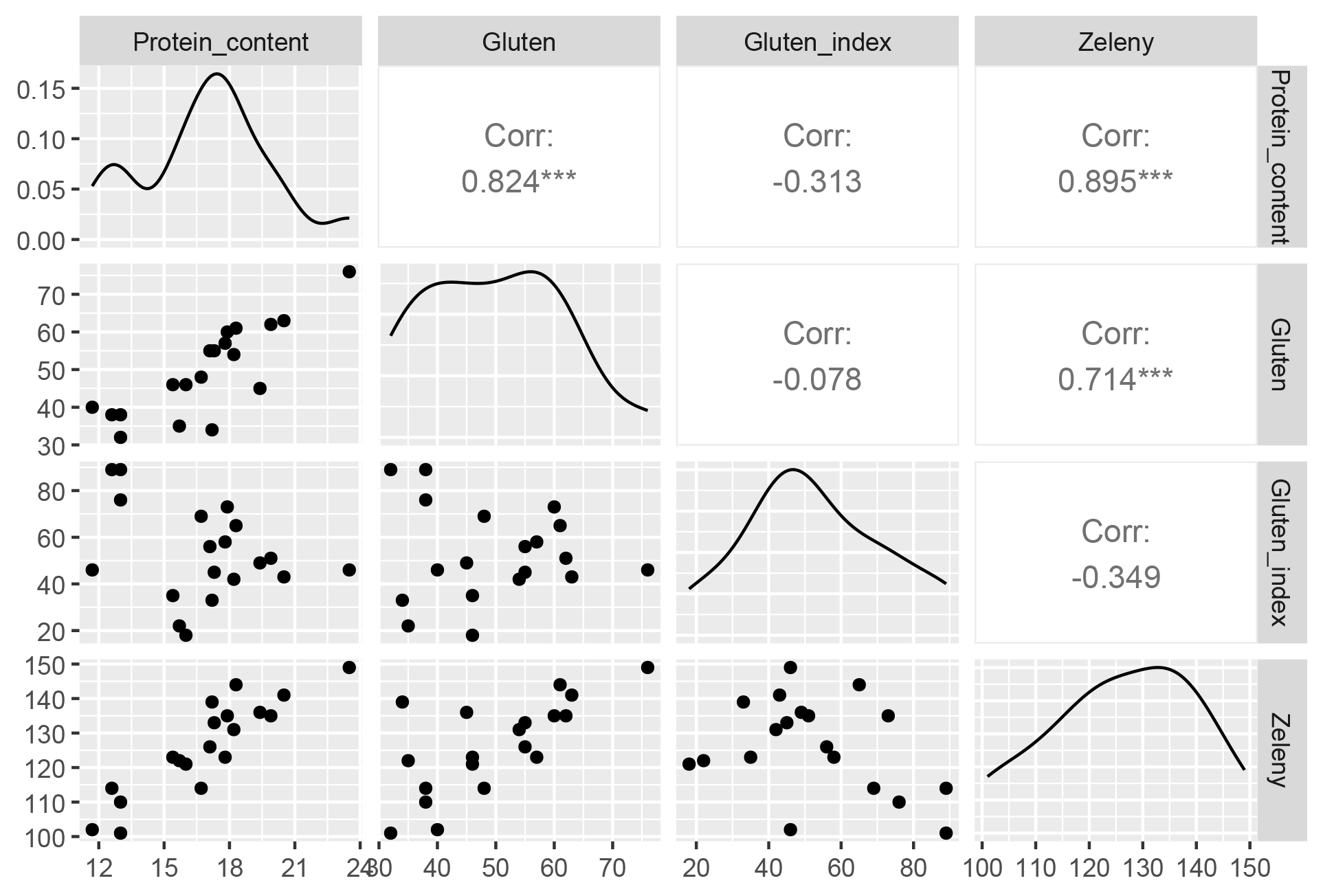

Supplement: S4 Fig — (TIF) [file pone.0331619.s009.tif]
